# Metformin versus chromium picolinate in clomiphene citrate-resistant patients with PCOs: A double-blind randomized clinical trial 

**Published:** 2013-08

**Authors:** Sedigheh Amooee, Mohammad Ebrahim Parsanezhad, Maryam Ravanbod Shirazi, Saeed Alborzi, Alamtaj Samsami

**Affiliations:** *Department of Obstetrics and Gynecology, Shahid Faghihi Hospital, Shiraz University of Medical Sciences, Shiraz, Iran.*

**Keywords:** *Chromium picolinate*, *Metformin*, *Polycystic ovary Syndrome (PCOS)*, *Clomiphen resistant*.

## Abstract

**Background:** Chromium picolinate could be effective in clomiphen citrate resistant PCOS patients.

**Objective:** To compare the effects of chromium picolinate vs. metformin in clomiphen citrate resistant PCOS patients.

**Materials and Methods:** The present randomized clinical trial was performed on 92 women with clomiphen citrate-resistant PCOS at the clinics which were affiliated to Shiraz University of Medical Sciences, Shiraz, Iran. The subjects were randomly assigned to two groups receiving either chromium picolinate (200µg daily) or metformin (1500mg daily) for 3 months. Anthropometric and hormonal profile were measured and compared both before and after the treatment. Ovulation and pregnancy rate was measured in the two study groups, as well.

**Results:** Chromium picolinate significantly decreased fasting blood sugar (FBS) after 3 months of treatment (p=0.042). In the same way, the serum levels of fasting insulin had significantly decreased leading to an increase in insulin sensitivity as measured by QUICKI index (p=0.014). In comparison to the patients who received chromium picolinate, those who received metformin had significantly lower levels of testosterone (p=0.001) and free testosterone (p=0.001) after 3 months of treatment. Nevertheless, no significant difference was found between the two study groups regarding ovulation (p=0.417) and pregnancy rates (p=0.500).

**Conclusion:** Chromium picolinate decreased FBS and insulin levels and, thus, increased insulin sensitivity in clomiphene citrate-resistance PCOS women. These effects were comparable with metformin; however, metformin treatment was associated with decreased hyperandrogenism. Overall, chromium picolinate was better tolerated compared to metformin; nonetheless, the two study groups were not significantly different regarding ovulation and pregnancy rates.

**Registration ID in IRCT: **IRCT201203139281N1

## Introduction

Polycystic Ovary Syndrome (PCOS) is a form of functional ovarian hyperandrogenism which affects approximately 5-10% of the women in the reproductive age (1). It is the most prevalent cause of female infertility and is characterized by ovarian hyperandrogenism and chronic anovulation. Insulin resistance with compensatory hyperinsulinemia appears to be the most universal feature of the polycystic ovarian disease and has a pathophysiologic role in the hyperandrogenism of the disorder (2). Nearly 20% of obese women with PCOS have an impaired Glucose Tolerance Test (GTT) or diabetes (1). Insulin sensitivity is impaired in PCOS and this finding holds in both the presence and absence of obesity (3). Evidence from in vivo and in vitro studies suggests that insulin has both direct and indirect effects on androgen levels. 

Moreover, ovaries removed from the women with PCOS exhibited enhanced androstenedione and testosterone release in response to insulin stimulation (1). Furthermore, it has been shown that acute increment in insulin levels in the women with PCOS induces rises in androgen levels (2). On the other hand, a reduction in serum insulin through the use of diazoxide to supress insulin secretion is reported to significantly reduce the serum testosterone (1, 2). The therapies which aimed at reducing hyperinsulinemia or insulin resistance in PCOS have resulted in slight amelioration of hyperandrogenism and the metabolic abnormalities of the syndrome. Metformin (MTF) is a drug which is routinely used for its antidiabetic effects on non-insulin-dependent diabetes mellitus (1, 4). 

In addition, MTF is claimed to have a multifactorial action with prime effects on insulin sensitivity in both liver and the peripheral tissue (1, 2). Besides, it is antihyperglycemic in action and does not cause clinical hypoglycemia (1). A recent study has shown some improvement in insulin resistance following the use of chromium (5). Chromium picolinate, an over-the-counter product, improved insulin sensitivity dietary at the insulin receptor level and, at the elevated level of intake, was devoid of adverse effects in human studies. 

Chromium picolinate consists of trivalent chromium, an extremely safe and highly tolerable trace mineral which is present in normal diet and is combined with picolinate acid in order to enhance gut absorption (5). Chromium picolinate effectively reduced insulin resistant and treated hyperinsulinemia as well as hyperandrogenemia but did not significantly affect the hormonal changes (4). In the women with PCOS, chromium picolinate (200 µg daily) improved the glucose tolerance but did not improve ovulation or hormonal profiles (3). Thus, the present randomized clinical trial aims to compare the effect of combination of clomiphene + metformin and clomiphene + chromium picolinate on ovulation induction and pregnancy rate in clomiphene citrate-resistant patients with PCOS. 

The effects on clinical as well as para-clinical abnormalities and BMI (body mass index) are also going to be determined. 

## Materials and methods

**Study population**

The current randomized clinical trial was performed in Mother and Child Hospital, a tertiary healthcare center affiliated to Shiraz University of Medical Sciences, Shiraz, Iran, during a 15-month period from August 2009 to January 2011. The study protocol was approved by the Institutional Review Board (IRB) of Shiraz University of Medical Sciences, Shiraz, Iran and the approval of the Ethics Committee was also achieved before beginning the study. In addition, written informed consents were obtained from all the participants. 

The study was conducted on 92 patients diagnosed with clomiphen citrate-resistance PCOS suffering from infertility who had referred to the infertility clinics of Mother and Child hospital medical assistance. PCOS was defined according to Rotterdam European Society for Human Reproduction and Embryology (ESHRE)/ American Society for Reproductive Medicine (ASRM) PCOS consensus workshop (6). All the patients had at least two of the three following criteria: I) chronic anovulation, II) clinical and/or biochemical evidence of androgen excess, and III) polycystic-appearing ovaries on transvaginal ultrasound. 

Clomiphen-resistance was defined as failure to have an ovarian response for 3 consecutive cycles in vaginal sonography after treatment with 150 mg/dayclomiphen (CC) from the fifth to the ninth day of each cycle. It should be noted that none of the subjects had taken any medication which could influence carbohydrate metabolism, including oral contraceptives, from 2 months before the onset of the study. Diabetic patients with a fasting glucose of <120 mg/dL were excluded from the study. The patients with Cushing’s syndrome, hyperprolactinemia, diabetes mellitus (DM), thyroid disease, adrenal hyperplasia, and androgen-secreting tumors or other endocrinopathies were excluded from the study, as well. 

Besides, the patients with adrenal hyperplasia were excluded by ACTH-stimulated 17-hydroxyprogesterone levels less than 10 ng/ml, and ACTH-stimulated 11-deoxycortisol levels less than 21 ng/ml of a historical control group of 60 healthy controls] (7, 8). The patients who smoked as well as those with kidney or liver diseases and breast cancer were also excluded from the study. None of the participants received oral contraceptives (OCPs), steroid hormones, or any medications which could interfere with lipid metabolism, ovarian, pituitary, and hypothalamic function, or insulin sensitivity during the last 3 months before the study. All the patients followed almost the same exercises and diet protocols during the study period. Besides, all the subjects had normal physical activity and did not drink alcoholic beverages. 

**Study protocol**

On the third day of a spontaneous or progesterone-induced menstrual cycle, after an overnight fast, blood samples were taken in order to determine serum follicular-stimulating hormone (FSH), luteinizing hormone (LH), total testosterone (T), free testosterone (fT), prolactin (PRL), thyroid stimulating hormone (TSH), fasting blood sugar (FBS), and fasting insulin. Moreover, oral glucose tolerance test was performed by Auto-analyzer method. Insulin sensitivity was assessed through quantitative insulin sensitivity check index (QUICKI) defined as 1/[log(fasting insulin)+log(fasting glucose)]. Finally, body mass index (BMI, as weight in kilograms divided by height in meters squared) was calculated by a single physician who was blinded to the study groups. 

**Figure 1:**
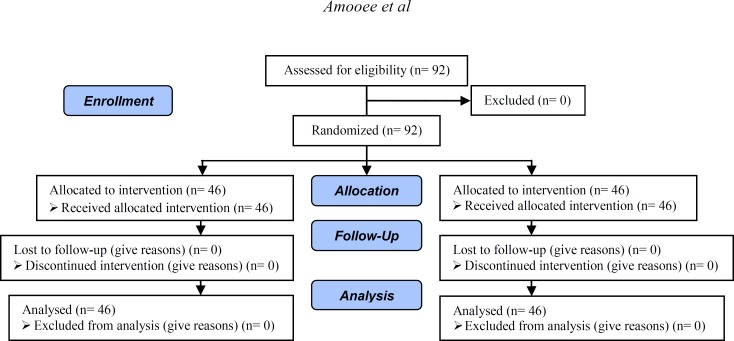
Consort flow diagram

All the patients were visited by a physician blinded to the study on the first day of the study and were given a sealed envelope including their registration number based on the arrival order. Then, using a computer-based random digit generator (Figure 1), the patients were randomly assigned to two groups receiving either 200µg chromium picolinate (Chromium picolinate American 21^st^ century medical industries USA) every day (n=46) or two tablets of the placebo or metformin (Apometformin Canadian Apotex medical industries Canada) three times a day (n=46). 

The study groups were parallel and matching was done. Moreover, the sample size was approved by a statistical advisor according to the previous researches conducted on the issue. Chromium picolinate or metformin were given without interruption between the cycles. The measurements were repeated after 3 months of treatment. All the patients received100mg clomiphen (Clomid Iran hormone medical industries Iran) from the 5^th^-9^th^ day of the cycle every day. Of course, clomiphen was given just for one cycle. Transvaginal ultrasonography was performed using Schimatzo with 7.5 Mega Hertz probe. All sonographies were performed in IVF Department of Mother and Child Hospital for follicle monitoring by one sonographist.

Serum progesterone level was checked on the 21^st^-23^rd^ cycle days. If at least 1 follicular size was larger than 18 mm or serum progesterone level was larger than 3ng/ml, ovulation induction had occurred. In case of missed menstrual period, β-HCG was checked for detection of pregnancy 1 week after the missed period. If β-HCG level was >25 (by the vidas method), the patient was pregnant and medications were discontinued. It should be noted that ultrasonographic evaluation and drug administration was performed by individual persons. 

All the hormonal assays were performed in Mother and Child Hospital Laboratory Center. Serum FSH (FSH-IRMA, KIP0264, BIOSOURCE, Nivelles, Belgium), LH (LH-IRMA CT, REF KP7CT, RADIM, Rome, Italy), PRL (PRL-IRMA, KIP1406, BIOSOURCE, Nivelles, Belgium), and TSH (TSH-IRMA, KIP1534, BIOSOURCE, Nivelles, Belgium) were measured through immunoradiometric assay. In addition, serum testosterone and free testosterone levels were measured by radioimmunoassay (EIA72K2, 10 Monobind, USA). Finally, serum levels of insulin were assessed by enzyme-linked immunosorbent assay (ELISA) (EIA24K8, 10 Monobind, USA). The intra-assay and interassay coefficients of variation were <6% for all the assays. Sonographists, patients, and the physician who visited the patients were blinded to the study. The drugs' side effects, including abdominal discomfort, vomiting, diarrhea, indigestion, headache, nausea, and loss of appetite, were asked and recorded.

**Statistical analysis**

Based on 90% power to detect significant differences between the corresponding variables (p=0.05, 2-sided), 40 patients were required in each group. Of course, in order to compensate for the possible nonevaluable data, 46 participants were enrolled in each group. All the statistical analyses were performed through the statistical software package SPSS for Windows, version 16.0 (SPSS, Chicago, IL, USA). Paired t-test was used in order to compare the results within groups, while independent t-test was utilized for comparing the results between the groups. In addition, the proportions were compared using χ^2^ test. 

The data were reported as mean±SD and p<0.05 was considered as statistically significant. 

## Results

A total of 92 patients with clomiphene citrate PCOS resistance were enrolled into the present study and divided into two groups, each including 46 patients. All the patients continued the follow up because the researcher was responsible their questions and followed them. The mean age of the patients was 26.9±5.1 (range 18-38) years. The baseline characteristics of the study subjects are presented in table I. As can be seen, no significant difference was found between baseline anthropometric and hormonal measurements. Table II summarizes the anthropometric and hormonal measurements before and after 3 months of treatment with chromium picolinate or metformin. In the patients who received chromium picolinate, FBS significantly decreased after 3 months of treatment (p=0.042).

In the same way, the serum levels of fasting insulin significantly decreased leading to an increased insulin sensitivity as measured by QUICKI index (p=0.014). On the other hand, BMI significantly decreased in those who received metformin for 3 months (p=0.041). In the same way, serum levels of testosterone (p=0.002), free testosterone (p=0.031), FBS (p=0.031), and fasting insulin level (p=0.001) significantly decreased leading to an increased insulin sensitivity assessed by QUICKI index (p=0.029).

Abnormal GTT test also significantly decreased in both study groups. However, no significant difference was observed between the two study groups after 3 months of treatment (p=0.163). After 3 months of treatment by chromium picolinate, serum levels of testosterone decreased by 0.12, while it decreased by 0.14 in those who received metformin (p=0.001). In the same way, the serum levels of free testosterone decreased by 0.2 and 1.1 in chromium and metformin groups, respectively and the difference was statistically significant (p=0.001). No other differences were found between these two treatment methods. Table III demonstrates the changes in the baseline values of the patients after 3 months of treatment. 

Overall, the patients who received metformin experienced more side effects compared to those receiving chromium picolinate (p=0.001). Moreover, metformin administration was accompanied by higher incidence of abdominal discomfort, nausea, vomiting, diarrhea, and indigestion, while chromium picolinate was accompanied by loss of appetite and headache (Table IV). Among those who received chromium picolinate, 22 (47.8%) ovulated during the study period and 9 (19.6%) conceived. Also, 20 (43.5%) patients of the metformin group ovulated and 10 (21.7%) conceived during the study period. Nevertheless, no significant difference was observed between the two study groups regarding the ovulation (p=0.417) and pregnancy rates (p=0.500) (Table V).

**Table I:** Baseline characteristics of the two study groups

Variable	Chromium picolinate	Metformin	p-value
Age (years)	26.8 ± 3.6	26.4 ± 5.3	0.752
Infertility duration (years)	2.6 ± 1.9	2.7 ± 1.1	0.904
BMI (kg/m^2^)	28.52 ± 1.61	29.24 ± 1.11	0.078
Testosterone (ng/ml)	0.86 ± 0.07	0.83 ± 0.09	0.226
Free testosterone (ng/ml)	2.6 ± 1.3	2.5 ± 0.9	0.064
LH (mIU/ml)	9.26 ± 1.17	9.41 ± 1.1	0.174
FSH (mIU/ml)	6.68 ± 1.08	6.67 ± 0.94	0.514
LH/FSH	1.33 ± 0.37	1.35 ± 0.38	0.271
TSH (µg/dL)	3.35 ± 1.5	3.22 ± 2.6	0.362
Prolactin (ng/dL)	171.5 ± 123.4	167.3 ± 112.9	0.073
FBS (mg/dL)	84.5 ± 8.88	83.86 ±9.27	0.092
Fasting Insulin (µU/ml)	17.17 ± 4.03	16.2 ± 3.65	0.144
QUICKI	0.317 ± 0.008	0.319 ± 0.007	0.081
Abnormal GTT (%)	2 (4.3%)	1 (2.2%)	0.500

Values are expressed as the mean ± SD. Significant difference between two groups (p<0.05)

BMI: Body mass index

GTT: Glucose tolerance test

FBS: Fasting blood glucose

FSH: Follicular stimulating hormone

LH: Luteinizing hormone

QUICKI: Quantitative insulin sensitivity check index

TSH: Thyroid stimulating hormone

**Table II:** Hormonal and metabolic parameters in both groups before and after 3 months of treatment

Variable (n=46)	Chromium picolinate		p-value	Metformin		p-value
	Before (n=46)	After (n=46)		Before (n=46)	After	
BMI (kg/m^2^)	28.52 ± 1.61	27.31 ± 1.58	0.086	29.24 ± 1.11	27.07 ± 1.57	0.041
Testosterone (ng/ml)	0.86 ± 0.07	0.74 ± 0.1	0.151	0.83 ± 0.09	0.69 ± 0.1	0.002
Free testosterone (ng/ml)	2.5 ± 0.9	2.3 ± 0.8	0.295	2.6 ± 1.3	1.4 ± 1.3	0.031
LH (mIU/ml)	9.26 ± 1.17	8.34 ± 1.39	0.168	9.41 ± 1.1	8.78 ± 1.27	0.312
FSH (mIU/ml)	6.68 ± 1.08	6.52 ± 0.94	0.078	6.67 ± 0.94	6.19 ± 0.81	0.095
LH/FSH	1.33 ± 0.37	1.27 ± 0.22	0.093	1.35 ± 0.38	1.41 ± 0.3	0.106
TSH (µg/dL)	3.35 ± 1.5	2.98 ± 1.3	0.078	3.22 ± 2.6	3.38 ± 1.8	0.235
Prolactin (ng/dL)	171.5 ± 123.4	226.8 ± 128.6	0.062	167.3 ± 112.9	222.52 ± 139.9	0.051
FBS (mg/dL)	84.5 ± 8.88	76.2 ± 16.92	0.042	83.86 ± 9.27	75.2 ± 13.82	0.031
Fasting insulin (µU/ml)	17.17 ± 4.03	14.9 ± 3.87	0.001	16.2 ± 3.65	14.69 ± 2.78	0.001
QUICKI	0.317 ± 0.008	0.328 ± 0.016	0.014	0.319 ± 0.007	0.328 ± 0.005	0.029
Abnormal GTT (%)	2 (4.3%)	1 (2.2%)	0.03	1 (2.2%)	0 (0%)	0.01

Paired t-Test. Values are expressed as the mean ± SD. Significant change from baseline in each group (p<0.05).

BMI: Body mass index

FBS: Fasting blood glucose

FSH: Follicular stimulating hormone

GTT: Glucose tolerance test

LH: Luteinizing hormone

QUICKI: Quantitative insulin sensitivity check index

TSH: Thyroid stimulating hormone

**Table III:** Changes in the baseline values of 92 PCOS patients after 3 months of treatment with chromium picolinate and metformin.

Variable	Chromium (n=46)	Metformin (n=46)	p-value
BMI (kg/m^2^)	-2.21 ± 1.51	-2.17 ± 1.02	0.341
LH (mIu/ml)	-0.92 ± 0.91	-0.63 ± 0.75	0.081
FSH (mIu/ml)	-0.16 ± 0.61	-0.48 ± 0.63	0.429
LH/FSH	-0.06 ± 0.02	0.06 ± 0.24	0.862
Testosterone (ng/ml)	-0.12 ± 0.06	-0.14 ± 0.038	0.001
Free testosterone (ng/ml)	-0.2 ± 0.1	-1.1 ± 0.4	0.001
TSH (µg/dL)	-0.037 ± 0.01	0.16 ± 0.02	0.068
Prolactin (ng/dL)	55.3 ± 112.3	55.22 ± 109.6	0.816
FBS (mg/dl)	-8.3 ± 11.76	-8.66 ± 16.3	0.648
Fasting Insulin (mIu/ml)	-2.26 ± 2.62	-1.50 ± 2.81	0.222
QUICKI	0.011 ± 0.002	0.009 ± 0.004	0.084

Independent student *t* Test. Values are expressed as the mean ± SD Significant change from baseline in each group (p<0.05).

BMI: Body mass index

FBS: Fasting blood glucose

FSH: Follicular stimulating hormone

LH: Luteinizing hormone

QUICKI: Quantitative insulin sensitivity check index

**Table IV:** Side effects of chromium picolinate and metformin in 92 patients with clomiphen citrate resistant PCOS

	Chromium picolinate	Metformin	p-value
Abdominal discomfort (%)	2 (4.3%)	11 (26.2%)	0.002
Nausea (%)	2 (4.3%)	7 (15.2%)	0.028
Vomiting (%)	0 (0%)	2 (4.3%)	0.001
Diarrhea (%)	0 (0%)	3 (6.56%)	0.001
Indigestion (%)	0 (0%)	2 (4.3%)	0.039
Loss of appetite (%)	4 (8.7%)	1 (2.1%)	0.018
Headache (%)	3 (6.56%)	0 (0%)	0.001
Total (%)	11 (23.9%)	26 (56.2%)	0.001

χ^2^ test.

**Table V:** Ovulation and pregnancy rates in 92 patients with PCOS who underwent 3 months treatment with chromium and metformin

	**Chromium picolinate**	**Metformin**	**p-value**
Ovulation rate (%)	22 (47.8%)	20 (43.5%)	0.417
Pregnancy rate (%)	9 (19.6%)	10 (21.7%)	0.500

χ^2^ test.

## Discussion

Although there is no direct evidence of chromium deficiencies in humans, dietary supplements exist to provide supraphysiological doses of absorbable chromium^3+^. Chromium^3+^ may act clinically by interfering with iron absorption, decreasing the high iron stores that are linked to diabetes and heart disease (9). Chromium functions as a part of an auto amplification system for insulin signaling and promotes enhancement of insulin sensitivity (10).

Studies suggest that chromium deficiency may increase the risk of cardiovascular diseases, elevate the blood glucose, lipid, and insulin level, and decrease the BMI (11). In our study, a significant decrease was observed in BMI in the group receiving metformin (group A) (p=0.041). In the study by Kazerooni *et al* 500 mg Metformin was used three times a day (12). In that study, the decrease in BMI was completely overt in the patients with normal Dehydroepiandrosterone sulfate (DHEAS); however, DHEAS was not considered in our study. In contrast, no decrease in BMI was observed in PCOS patients in the study by Genazzani *et al* which used 500 mg metformin two times a day for 6 months (13). 

This difference might be due to the variation in the BMI of Genazzani's and the present study's patients. Ethnic differences could play a role, as well. Aruna *et al* also conducted a study using 500 mg metformin two times a day in 50 patients and reported a decrease in BMI (BMI=25) (14). In the group in which chromium was used, no significant change was found in BMI before and after the therapy. In the same line, in the study by Lydic *et al* 1000 µg chromium was used daily in PCOS patients for 2 months, but no significant change was found in BMI (5). Another study (Anderson) also reported no changes in body composition after receiving chromium (15). 

Furthermore, in another study by Albarracin *et al* using 600 µg chromium in diabetes mellitus type 2 patients had no significant effect on BMI (16). In the present study, a decrease was reported in total and free testosterone in metformin group. On the other hand, no changes were observed in total testosterone level in the study by Aruna *et al* (14), which could be due to ethnic differences as well as different dosages used in the two studies. Different methods used for selecting the patients could have played a role, as well. Similar results were also obtained by Genazzani *et al* who had conducted their study on non-obese patients (13). 

The decreasing effect of metformin on testosterone was also reported by kolodzieyczyk *et al* (17). In the chromium group, on the other hand, no change was found in total and free levels of testosterone. Similar results were also obtained by Lydic *et al* and Lucidi *et al* (3, 5). However, we cannot provide any explanation for the effect of chromium on testosterone. Metformin is known to decrease FBS, fasting insulin, QUICKI index, and abnormal GTT and similar results were also achieved in our study (17). On the contrary, these findings were not reported by Genazzani *et al* which could be due to the low body weight of their study patients (13). In Aruna's study also, no decrease was revealed in FBS, fasting insulin, QUICKI, and abnormal GTT, while Kazerooni *et al* showed using metformin to be effective in decreasing FBS (12, 14).

The findings of the present study showed changes in FBS, fasting insulin, QUICKI, and abnormal GTT in the chromium group, which is in contrast with the results obtained by Lydic *et al* which might be due to the high BMI of the selected patients. In the study conducted by Lucidi *et al* no changes were found in FBS and insulin sensitivity, while GTT had changed; of course, only 6 patients took part in that study. Albarracin *et al* performed a study on obese patients with diabetes mellitus type 2 and reported a decrease in FBS which is consistent with the findings of the current study. 

A large number of studies have confirmed the effectiveness of chromium in decreasing FBS, HbA1C, and fasting and 2 hours insulin in the patients with diabetes mellitus type 2 (10). Metformin has the same effect as chromium on the pregnancy rate. It seems this study is the first research comparing the effect of metformin and chromium on pregnancy rate. The effect of metformin on regulation of the menstrual period has also been reported in different studies (12). In Aruna's study, using metformin improved ovulation and the pregnancy rate. Also, regulation of menstruation was reported in the studies conducted by Genazzani *et al* and Kolodzieyczyk *et al* (13, 17). 

Lucidi *et al* used 200 microgram chromium in 6 patients in order to investigate the effect of chromium on ovulation. The study results revealed regulation in menstruation which could be a sign of ovulation (3). However, the menstrual period was not taken into account in our study. Of course, in line with the study by Albarracin *et al* chromium had very low side effects. Due to the low side effects and having equal effects on the pregnancy rate, metformin could be replaced by chromium in some PCOS patients (16). 

One of the limitations of the present study was the absence of a control group which is better to be considered in future studies. Furthermore, using a larger number of patients in both groups and evaluating the patients with various dosages of metformin and chromium can be helpful for finding the optimum dosage of chromium and substituting chromium with Metformin.

## Conclusion

In conclusion, chromium picolinate decreased FBS and insulin levels and, thus, increased insulin sensitivity in clomiphen citrate-resistance PCOS women. However, chromium picolinate treatment was not associated with any other effects on other hormonal profiles, including testosterone. These effects were comparable with metformin; however, metformin treatment was associated with decreased hyperandrogenism. Overall, chromium picolinate was better tolerated than metformin; nevertheless, no significant differences were observed between the two groups regarding ovulation and pregnancy rates.
